# Feasibility and safety of electrohydraulic acoustic therapy for treatment of hypertension in patients with chronic kidney disease

**DOI:** 10.3389/fmedt.2026.1735319

**Published:** 2026-03-03

**Authors:** Aleksandra Kukla, Alex Slutzker, Alexandra Perez Alterman, Petar Veruovic, Michael Glikson, Shuli Silberman, Amir Lerman, Lilach O. Lerman, Talya Wolak

**Affiliations:** 1 Division of Nephrology and Hypertension, Department of Medicine, Mayo Clinic, Rochester, MN, United States; 2 Curespec LTD, Yehud-Monosson, Israel; 3 Medistat Ltd., Tel Aviv, Israel; 4 Faculty of Medicine Hradec Králové, Charles University, Hradec Králové, Czechia; 5 Jesselson Integrated Heart Center Shaare Zedek Medical Center, Faculty of Medicine, Hebrew University, Jerusalem, Israel; 6 Department of Cardiovascular Diseases, Mayo Clinic, Rochester, MN, United States; 7 Internal Medicine Division, Shaare Zedek Medical Center, Faculty of Medicine, Hebrew University, Jerusalem, Israel

**Keywords:** ambulatory blood pressure monitoring, antihypertensive therapy, blood pressure, chronic kidney disease, electro-hydraulic acoustic therapy, hypertension, device-based therapy

## Abstract

**Background:**

Uncontrolled hypertension is common in chronic kidney disease (CKD) patients. Electro-hydraulic acoustic therapy (eHAT) is a non-invasive treatment that may lower blood pressure (BP), but its safety and efficacy in CKD remain unclear. This study aimed to evaluate eHAT's feasibility and safety in this population.

**Methods:**

In this single-arm, prospective, single-center proof-of-concept study, CKD patients received six eHAT treatments over three weeks. BP was assessed in-office (attended and unattended) at baseline and at 4-, 12-, 24-, and 48-weeks post-treatment. Ambulatory BP monitoring (ABPM) was performed at baseline, 12, and 48 weeks. The primary endpoint was change in systolic office BP (OBP) from baseline to 12 weeks. Secondary endpoints included additional BP measurements, kidney function, and safety.

**Results:**

Fifteen patients completed all follow-ups. At 12 weeks, mean systolic OBP decreased by 9.73 mmHg (SD 14.77; *p* = 0.0032) and remained significantly lower at 24 weeks (−7.67 mmHg; *p* = 0.0335) and 48 weeks (−19.40 mmHg; *p* < 0.0001). Diastolic OBP reductions were significant at 12 (*p* = 0.0413) and 48 weeks (*p* = 0.0022). By 12 weeks, 26% of participants reduced the number or dose of antihypertensive medications. Kidney function remained stable throughout. No safety signals were detected.

**Conclusion:**

Our study highlights the feasibility and tolerability of eHAT in patients with CKD and HTN. Nonetheless, due to the small sample size and lack of a control group, these findings should be considered preliminary.

## 1 Introduction

Hypertension (HTN) and chronic kidney disease (CKD) are pressing global health concerns, with increasing prevalence rates and significant implications for morbidity and mortality (1, 2). Moreover, patients with CKD frequently face the challenge of polypharmacy, with 48% taking 5–9 medications, and 29% ≥ 10 medications (3). Over 30% of patients with CKD have poor adherence to antihypertensive therapy, which is associated with poor blood pressure (BP) control (4). Therefore, there is an urgent need for hypertension treatments that are safe for use in individuals with CKD, effective in managing BP, do not adversely affect kidney function or electrolyte balance (e.g., hyperkalemia), can be administered under medical supervision, and do not add to the pharmaceutical treatment burden.

One of the emerging alternatives to pharmacotherapy are device-based interventions such as renal denervation (RDN) (5), baroreflex activation therapy (BAT) (6) or cardiac neuromodulation (7). RDN is now considered as an option for patients with true resistant hypertension (RH), as well as patients who are nonadherent and/or intolerant to medical therapy (5). While successful, these procedures are invasive, require skilled and well-trained operators, and rely on iodine contrast administration directly to the kidney, raising concerns of nephrotoxicity in patients with CKD.

Electro-hydraulic acoustic therapy (eHAT) represents a promising frontier in HTN management, offering a non-invasive, non-pharmaceutical, ambulatory, and potentially transformative approach to modulating BP (8). eHAT delivers an acoustic wave characterized by an abrupt spike; this type of wave exhibits high peak pressure, typically up to 19.6 MPa (Nephrospec user manual) and a short life cycle lasting approximately 1 µs. The specific energy parameters for eHAT were selected based on preclinical studies demonstrating that low-intensity shock waves at 0.09 mJ/mm^2^ with 200–300 impulses optimize cellular viability, enhance expression of angiogenic factors, and minimize apoptosis, thereby promoting tissue repair without detrimental effects (9, 10). eHAT targets tissues directly, using electro-hydraulically generated acoustic waves to induce mechanotransduction in renal and vascular structures. It has been postulated that this therapy may stimulate nitric oxide signaling, microvascular remodeling, and better renal perfusion, all without nerve or parenchyma damage—especially important in CKD. In contrast, renal denervation lowers BP via thermal ablation of sympathetic nerves (11, 12). However, the extent to which eHAT therapy reduces BP in individuals with CKD and its safety in terms of the kidney function in this high-risk population remains unknown.

To fill this gap in the literature, we tested the hypothesis that eHAT reduces BP while maintaining renal function in hypertensive individuals with CKD in the pilot prospective clinical cohort study.

## 2 Methods

### 2.1 Study design

The study was an interventional, prospective, single-arm, single center proof of concept trial for patients diagnosed with HTN and CKD stage 3 and 4, as defined by the estimated glomerular filtration rate (eGFR) between 20 and 60 mL/min/1.73m^2^ as per the CKD-EPI equation 2021 (13). Screening, enrollment, therapy and follow-up visits were performed at Sha’are Zedek Medical Center, Jerusalem, Israel. The protocol was approved by all local ethics committees and all patients provided written informed consent to participate in the trial. The trial was designed in accordance with the Declaration of Helsinki. An independent data safety and monitoring committee monitored patients for adverse events. The trial was registered on the Israeli clinical trials website (clinicaltrial.health.gov.il/clinicaltrials/?MOHResearchId=MOH_2021-12-29_010422) with trial identifier and registry number MOH_2021-12-29_010422.

### 2.2 Patients

Eligible patients aged between 18 and 80 years who carried a diagnosis of HTN and CKD were enrolled. Patients were excluded if they had a history of type-1 diabetes mellitus (DM), poorly controlled type-2 DM (HbA1c > 10%), anemia with hemoglobin (Hb) levels ≤ 9 grams per deciliter, macrohematuria, HTN secondary to an identifiable and treatable causes (other than known renal artery stenosis), were taking medications that can increase BP, or had active malignancy (primary tumor or metastasis). Other exclusion criteria were use of vitamin K antagonists or direct oral anticoagulants, abnormal platelets levels, history of hematologic disorders that increase the risk of bleeding; active pyelonephritis; history of kidney stones, end-stage kidney disease, kidney transplant or pregnancy. All patients were white of mixed European-Middle Eastern descent.

### 2.3 Procedure

The patients received eHAT treatments six times over a 3-week period using the Nephrospec^TM^ device (Curespec LTD). The patients were placed in the prone position during treatment, and the kidneys were localized using a standard ultrasound (US) device. No analgesics were administered, but the patients were instructed to notify the staff if they felt any pain or discomfort during the treatment. A total of 8 treatment zones each 10 mm in diameter were chosen on each kidney, localized by US and delivered from the posterior approach with 300 shockwaves applied to each location for a total of 2400 shockwaves per kidney per treatment session. Both kidneys were treated (total of 4,800 shocks per treatment). Treatment energy was 0.09 mj/mm^2^, at a frequency of 160 shocks/min (2.66HZ). Patients were studied at the end of the treatment (EOT), 4, 12, 24 and 48 weeks after the last treatment (Supplementary Figure S1).

Patients had their attended office BP (OBP) and unattended office BP (uOBP) measured at every follow up visit; 24-hour ambulatory BP monitoring (ABPM) was obtained at the baseline, 12 weeks and 48 weeks. In addition, blood and urine samples were obtained for analysis of kidney function and proteinuria at every follow-up visit.

### 2.4 Outcomes

#### 2.4.1 BP endpoints

The primary efficacy endpoint was change in the systolic OBP from the baseline to 12 weeks post-treatment. Other efficacy endpoints included change in the systolic and diastolic OBP, uOBP and ABPM from baseline up to 48 weeks of follow up.

OBP was measured three times in the sitting position after ten minutes of rest (with the average of last two to be considered), in the presence of personnel. uOBP was defined as an automatic OBP measured five times (average of last three to be considered), at random intervals, in the sitting position after ten minutes of rest without the presence of personnel.

#### 2.4.2 Effect of eHAT on the number of anti-hypertensive medications and dose adjustments

We utilized Measurement Index for Antihypertensive Medication Burden previously proposed (Medindex-1) (14) in its modified form (MedIndex-2) to assess the treatment effect on the number and dose of antihypertensive medications. These tools quantify the burden of antihypertensive medication and assess changes in treatment intensity over time. MedIndex-1 calculates the sum of prescribed-to-standard dose ratios, weighted by drug class (14). MedIndex-2 multiplies this sum by the number of medications, capturing both dosage and the number of prescribed medications. MedIndex-2 was adapted from MedIndex-1 to allow a more detailed characterization of changes in antihypertensive medication use over the course of the study. While MedIndex-1 provides a general summary of medication burden, MedIndex-2 offers additional sensitivity to differences in medication intensity and regimen changes that may not be fully captured by a single index. Although no standalone methodological publications are available for MedIndex-2, this approach was used in the clinical evaluation of the Symplicity Spyral™ Renal Denervation System, which formed part of the FDA-approved Summary of Safety and Effectiveness Data. The use of both indexes in that regulatory context supports the feasibility and interpretability of MedIndex-2 for analyzing medication changes in trials where treatment effects and medication adjustments may occur simultaneously. https://www.accessdata.fda.gov/scripts/cdrh/cfdocs/cfpma/pma.cfm?id=P220026.

#### 2.4.3 Kidney endpoints

The impact of eHAT on kidney function was evaluated using serum creatinine and eGFR measurements at baseline and follow-up timepoints. The urine protein-to-creatinine ratio (uPCR) was also measured at these same time points.

Safety endpoints: any side effects reported by the patients that could be associated with the device or the protocol were recorded.

### 2.5 Statistical analysis

The study was designed to be descriptive by nature; no formal statistical significance tests will be applied. Nominal *p*-values will be reported for supplementary purposes.

All measured variables and derived parameters were summarized using descriptive statistics. Summary tables for categorical variables included sample size, absolute frequency, and relative frequency. For continuous variables, summary tables provided sample size, arithmetic mean, standard deviation, minimum, median, and maximum values.

Changes from baseline were calculated for each BP parameter, eGFR, and uPCR (with % change for uPCR). A mixed model for repeated measures was applied to each parameter change, adjusted for baseline values. One-sided *P*-values were presented in the tables for BP parameters. The null hypothesis assumes that BP will remain unchanged over time, whereas the alternative hypothesis predicts that BP will decrease. Since this is a one-sided hypothesis, the entire 5% type I error rate applies to one side, which leads to a one-sided *p*-value. For systolic BP parameters, the same model was used to determine if the decrease was greater than 5 mmHg. For uPCR, a mixed model for repeated measures was applied to % change. The data was analyzed using SAS® version 9.4 (SAS Institute, Cary, North Carolina).

## 3 Results

Between February 2022 and July 2023, we screened 24 patients, with 21 enrolled and 20 completing the eHAT regimen**.** Four patients subsequently dropped out of the study for various reasons before reaching the primary endpoint (unrelated surgery, inability to adhere to follow-up visit schedules). One patient was disqualified due to inadvertent errors in BP measurements and therefore inaccurate reading. Final analysis therefore included 15 subjects who completed study protocol (Supplementary Figure S2).

Table 1 depicts the baseline characteristics of the 15 analyzed subjects. Overall, there were more males than females, with a mean age (SD) of 69.5 (9) and 60% had diabetes. Fourteen subjects were on BP medications, and 5/15 (33%) had a history of RH. One subject who was not on anti-hypertensive therapy had OBP of 148/82 mmHg at baseline and 130/71 mmHg at the primary efficacy endpoint (12 weeks from EOT). None of the patients had renal artery stenosis. Mean OBP systolic (SD) and diastolic (SD) were 139.2 mmHg (15.7) and 77.4 mmHg (7.8) respectively. Mean eGFR (SD) was 45.6 (14) mL/min/1.73m^2^.

**Table 1:** Baseline characteristics of the study group.

Characteristics	*N* = 15
Age (years ± SD)	69.5 ± 9
Sex (*n*, %)	
Male	10 (67)
Female	5 (33)
BMI (kg/m²) (SD)	29.7 ± 5.93
BMI > 30 (*n*, %)	6 (40)
Race (%)	
White	15 (100)
Smoking (*n*, %)	
Never smoker	11 (73)
Former smoker	2 (13.5)
Smoker	2 (13.5)
Cardiovascular disease (%)	4 (27)
Diabetes mellitus (%)	9 (60)
Prescribed antihypertensive therapy (*n*, %)	14 (93%)
Antihypertensive medications (*n*, %)	
Diuretics	5 (33)
Beta blockers	9 (60)
Ace inhibitors	3 (20)
Angiotensin receptor blockers	9 (60)
Calcium channel blockers	13 (87)
Alpha blockers	6 (40)
Office blood pressure (mmHg, SD)	
Systolic	139.2 (15.7)
Diastolic	77.4 (8.67)
MAP	98 (7.80)
Protein to creatinine ratio (mg/g, SD)	681 (981.6)
eGFR (mL/min/m^2^) (SD)	45.60 (14.11)

SD, standard deviation; BMI, body mass index; eGFR, estimated glomerular filtration rate; MAP, mean arterial blood pressure.

### 3.1 Effects of eHAT on BP

**Figure 1:**
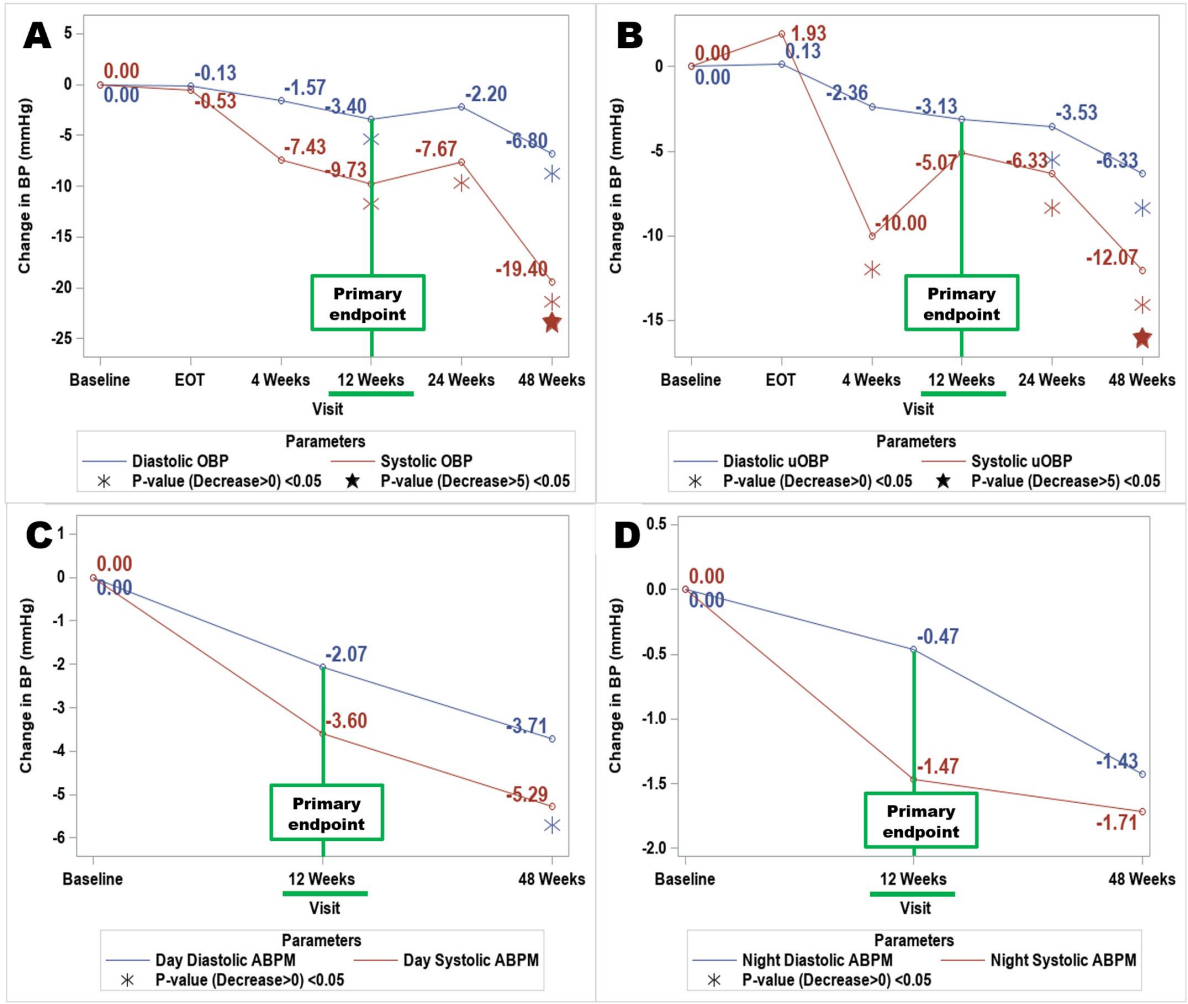
Change in blood pressure (attended vs. Unattended vs. ABPM day/night) post electro-hydraulic acoustic therapy: baseline to 48-week follow-up. **(A)** Attended office blood pressure; **(B)** unattended office BP; **(C)** Daytime Ambulatory Blood Pressure Monitoring; **(D)** Nighttime Ambulatory Blood Pressure Monitoring; OBP, Office Blood Pressure; uOBP, unattended office blood pressure; ABPM, Ambulatory Blood Pressure Monitoring; EOT, End of Treatment.

Table 2 lists BP values (OBP, uOBP, and ABPM) at baseline and follow-up visits, while Table 3 illustrates the changes in BP parameters from baseline to the follow-up time points. Changes in systolic and diastolic OBP and uOBP are graphically illustrated in Figure 1.

**Table 2:** Baseline and follow up blood pressure results.

BP variable	Baseline	EOT	*P*	4 weeks	*P*	12 weeks	*P*	24 weeks	*P*	48 weeks	*P*
Office BP systolic mmHg (SD)	139.2 (15.65)	138.67 (14.78)	0.481	129.57 (15.80)	0.050	129.47 (11.84)	0.003	131.53 (14.02)	0.033	119.80 (12.16)	<.0001
Office BP diastolic mmHg (SD)	77.4 (8.68)	77.27 (10.44)	0.470	76.43 (10.83)	0.309	74.0 (9.43)	0.041	75.2 (6.24)	0.103	70.6 (8.55)	0.002
uOBP systolic mmHg (SD)	130.67 (8.66)	132.6 (17.42)	0.659	120.86 (14.46)	0.016	125.6 (14.29)	0.093	124.33 (13.32)	0.046	118.6 (9.42)	<.0001
uOBP diastolic mmHg (SD)	76.53 (8.43)	76.67 (8.52)	0.526	74.36 (10.13)	0.134	73.40 (11.08)	0.09	73.0 (7.61)	0.038	70.2 (8.6)	0.001
ABPM Overall systolic mmHg (SD)	145.67 (9.67)	X	X	X	X	142.67 (13.21)	0.185	X	X	141.64 (14.01)	0.148
ABPM overall diastolic mmHg (SD)	76.33 (10.96)	X	X	X	X	74.47 (13.65)	0.214	X	X	73.21 (12.13)	0.059

BP, blood pressure; uOBP, unattended office blood pressure; EOT, end of treatment; ABPM, ambulatory blood pressure monitor; SD, standard deviation.

**Table 3:** Change in blood pressure parameters from baseline to follow up visits.

Parameter	Visit: weeks		Mean (SD)	StdErr	Median	*P*-value
Office Systolic BP	EOT		−0.53 (21.13)	5.46	−2.00	0.481
	4		−7.43 (19.19)	5.13	−5.50	0.050
	12		−9.73 (14.77)	3.81	−7.00	0.003
	24		−7.67 (18.96)	4.90	−4.00	0.033
	48		−19.40 (19.75)	5.10	−12.00	<.0001
Office Diastolic BP	EOT		−0.13 (10.79)	2.79	0.00	0.470
	4		−1.57 (10.97)	2.93	−3.00	0.309
	12		−3.40 (6.82)	1.76	−4.00	0.041
	24		−2.20 (9.02)	2.33	−2.00	0.103
	48		−6.80 (8.96)	2.31	−4.00	0.002
uOBP Systolic BP	EOT		1.93 (19.02)	4.91	−4.00	0.659
	4		−10.00 (14.76)	3.94	−11.00	0.016
	12		−5.07 (14.93)	3.86	−5.00	0.093
	24		−6.33 (15.36)	3.96	−5.00	0.046
	48		−12.07 (11.14)	2.88	−14.00	<.0001
	EOT		0.13 (7.50)	1.94	−1.00	0.526
uOBP Diastolic BP	4		−2.36 (8.97)	2.40	−5.50	0.139
	12		−3.13 (8.21)	2.12	−4.00	0.092
	24		−3.53 (8.48)	2.19	−3.00	0.038
	48		−6.33 (6.29)	1.62	−6.00	0.001
ABPM Systolic Daytime	12		−3.60 (15.01)	3.88	−3.00	0.149
	48		−5.29 (14.17)	3.79	−3.50	0.086
ABPM Diastolic Daytime	12		−2.07 (9.51)	2.46	−1.00	0.207
	48		−3.71 (7.15)	1.91	−3.50	0.041
ABPM Systolic Nighttime	12		−1.47 (13.32)	3.44	1.00	0.342
	48		−1.71 (16.89)	4.51	−0.50	0.348
ABPM Diastolic Nighttime	12		−0.47 (8.58)	2.21	3.00	0.412
	48		−1.43 (8.46)	2.26	−2.00	0.278
ABPM Systolic Overall	12		−3.00 (13.23)	3.42	−1.00	0.185
	48		−4.14 (14.10)	3.77	−3.00	0.147
ABPM Diastolic Overall	12		−1.87 (8.74)	2.26	0.00	0.214
	48		−3.43(7.31)	1.95	−4.00	0.059

ABPM, ambulatory blood pressure monitor; BP, Blood pressure; EOT, end of treatment; uOBP, Unattended office blood pressure.

At the primary efficacy endpoint (12 weeks from baseline), systolic OBP (SD) decreased by 9.73 mmHg (14.77) from baseline (*p* = 0.0032). The reduction in systolic OBP was evident at 4 weeks post-treatment, trending towards statistical significance (*p* = 0.05), and remained significant at subsequent follow-up visits, with mean (SD) decreases of 7.67 mmHg (18.96), *p* = 0.0335, and 19.40 mmHg (19.75), *p* < 0.0001 at 24 and 48 weeks, respectively. The decrease in diastolic OBP reached statistical significance only at 12 weeks (*p* = 0.0413) and 48 weeks (*p* = 0.0022). Systolic and diastolic uOBP also decreased post-treatment, achieving statistical significance at all follow-up visits except at 12 weeks post-treatment for systolic and at 4- and 12-weeks post-treatment for diastolic uOBP (Table 3; Figure 1).

Systolic and diastolic ABPM at follow-up visits showed minor changes relative to baseline (Table 3; Figure 1). Overall systolic ABPM (SD) changed by −3 mmHg (13.23) at 12 weeks and −4.0 mmHg (14.10) at 48 weeks; these changes were modest and did not reach statistical significance in the overall analysis, including nighttime measurements. There were no statistically significant differences found in overall diastolic ABPM. Daytime systolic BP showed reductions of −3.6 mmHg (15.01) at 12- weeks and −5.29 mmHg (14.17) at 48- weeks of follow up, which did not reach statistical significance (*p* = 0.086 for both time points). A statistically significant change was observed only for daytime diastolic BP at 48 weeks, which decreased by −3.7 mmHg (7.1; *p* = 0.0412).

### 3.2 Adjustment in anti-hypertensive regimen

Despite the recommendations for both subjects and healthcare providers to try to avoid altering BP medications, five subjects experienced changes to their hypertension medication regimen by the 12-week follow-up. Specifically, two patients had their dosages reduced, three discontinued one of their hypertension medications, and one patient had an increased dosage. Overall, MedIndex 2 demonstrated a significant reduction in the number and dose of antihypertensive medications at the 12-month follow-up (*p* = 0.029) (Supplementary Figure S3). Individual data (Supplementary Table S1) revealed that only one subject experienced an increased burden at this time point.

### 3.3 Safety outcomes

eGFR remained stable throughout the study (Supplementary Figure S4). Similarly, there was no significant change in urine protein excretion in up to 48 weeks of follow up (data not shown).

Subjects did not experience any significant study-related adverse events during eHAT treatment. Two subjects reported mild, self-resolving back pain after the treatments. We did not observe any significant adverse events during first 30 days and 6 months post treatment. One subject who reported back pain during and after the treatment was subsequently diagnosed with a herniated disc, which was unrelated to the intervention.

Adverse events that were not related to the intervention included urinary tract infection, COVID-19 infection and ST-elevation myocardial infarction at 27th week post intervention

## 4 Discussion

This study showed that eHAT therapy improved OBP in subjects with HTN and CKD, although ABPM did not confirm the results. Therapy was overall well tolerated without major safety concerns. Hypertension is highly prevalent in individuals with CKD and constitutes both a cause and consequence of CKD progression. Despite advancements in treatment, as many as 40% of patients with CKD have been shown to have apparent treatment-RH, defined as BP ≥140/90 mm Hg on ≥3 antihypertensives, or use of ≥4 antihypertensive drugs based on Chronic Renal Insufficiency Cohort (CRIC) data (15). Others showed that over half of patients with CKD may have apparent treatment-RH when the currently recommended by Kidney Disease Improving Global Outcomes goal of BP <120 (16) is applied, underscoring the urgent need for innovative therapeutic modalities. Many patients either do not tolerate or prefer to avoid multiple medications, and the complexity of treatment increases the likelihood of non-adherence to prescribed regimens (17). Procedures such as RDN, baroreflex activation therapy, and arteriovenous anastomosis can be effective, but may be too invasive for many patients with CKD, DM, or obesity (18). In contrast, eHAT presents a promising option, but it's safety and efficacy need to be established in the larger number of patients.

The reduction in the number and dosage of antihypertensive agents following eHAT therapy, as measured by MedIndex, along with the observed decrease in BP, supports the possible role of eHAT in BP control. Therefore, eHAT could be used alongside or instead of blood pressure medications for patients with uncontrolled hypertension if future randomized controlled trials confirm its effectiveness.

The use of this device may be particularly advantageous for individuals who struggle with adherence to therapy, experience side effects from BP medications, or face a high treatment burden (19) (20) (21).Currently, the possible ways that eHAT may reduce BP are mostly uncertain and based mainly on animal research, not studies involving humans. It is hypothesized that eHAT could reduce BP through denervation of the kidney by delivering energy to the renal nerves, in a manner analogous to the RDN procedures used by RECOR (19) (acoustic) and MEDTRONIC (radiofrequency) (20). Supporting this hypothesis, previous research in pigs has shown a decrease in renal norepinephrine release following eHAT treatment (21); however, these findings have not yet been demonstrated in humans.

OBP was lower than the baseline up to 48 weeks of follow up. Long term anti-hypertensive effect of RDN was also previously demonstrated (up to 36 months of follow up) (22, 23), and real world evidence confirmed sustained BP lowering effect up to 10 years post procedure (24). Further studies are required to clarify whether the lower OPB was truly related to eHAT therapy and the mechanisms behind sustained BP improvement in these subjects.

Our data showed that both attended and unattended OBP improved after eHAT therapy more than ABPM. These findings align with previous reports that antihypertensive treatment in hypertensive patients results in a greater reduction in OBP compared to ABPM. This effect is observed in both SBP and DBP, with the treatment effect captured by OBP being 36% greater for SBP and 33% greater for DBP compared to 24-hour measurements (25). The treatment's impact is influenced by several factors that may have a differential impact on OBP vs. ABPM. For example, the “white-coat effect” affects OBP but not ABPM, and “the regression to the mean” phenomenon (where extreme measurements tend to move closer to the average upon remeasurement) affects single or few OBP measurements more than the 24-hour average of day and night measurements (25). Measurement bias in OBP and absence of blinding may have also contributed to stronger in-office responses. Overall, the small number of participants in this study does not allow for subgroup analysis to further elucidate individual characteristics predictive or larger ambulatory daytime SBP response. Moreover, the results may be affected by natural fluctuations in BP and by the influence of participating in a clinical trial, as earlier randomized controlled trials on antihypertensive treatments have shown that even patients assigned to placebo experienced documented BP improvements (19).

Importantly, patients experienced either transient or no side effects. In particular, kidney function remained stable over time. Urinary protein excretion, an important prognostic factor for kidney function decline (26), showed no change, and we found no negative impact of eHAT on eGFR that remained stable for up to 48 weeks of follow up. Indeed, eHAT has been recently shown to be safe in patients with diabetic nephropathy (27). Whether and through which mechanism eHAT might slow down deterioration of kidney function has not been fully explored in the current pilot study.

This study has several limitations that should be acknowledged. It was non-randomized and did not include a sham-treated control group. The severity of HTN was not considered for enrollment, with recruited participants ranging from no medications (one patient) to RH. The BP regimen at baseline was not standardized, and anti-hypertensive medications were changed by primary care providers. In addition, the protocol requires a 3-week regimen that might be impractical for some patients. Strengths of the study include its prospective design and longitudinal follow-up of up to 48 weeks, but the results should be considered hypothesis generating and require confirmation. eHAT.

## 5 Conclusion

In conclusion, eHAT should be explored for treatment of HTN in patients with CKD. Efficacy cannot be firmly attributed to eHAT without randomized sham-controlled trials.

Supplementary Figure S1 Study design. Participants underwent a screening process to assess eligibility, followed by baseline evaluation prior to intervention. Eligible participants received six sessions of electro-hydraulic acoustic therapies. The end of treatment evaluation was conducted shortly after the last therapy session. Subsequently, follow-up assessments were scheduled at 4-, 12-, 24-, and 48-weeks post-treatment to monitor outcomes over time. In all follow-ups, office and unattended office blood pressure was measured. In every follow-up laboratory blood and urine samples were taken to evaluate eGFR and uPCR. At 12- and 48-weeks post treatment ambulatory blood pressure monitoring was conducted.

Supplementary Figure S2 Study recruitment flowchart. Flowchart depicting the recruitment and follow-up process of recruiting study participants. Of 24 patients screened, 21 consented to participate, while 3 failed screening. One patient revoked consent, leaving 20 who completed all eHAT assessments. Of these, 4 dropped out during follow-ups, resulting in 16 completing all follow-ups. One patient was removed due to errors in BP readings, yielding 15 patients with complete and usable results.

Supplementary Figure S3 Medindex. Change in MedIndex-2 post electro-hydraulic acoustic therapy treatment: baseline to 48-week follow-up. MedIndex is a method used to quantify the use of antihypertensive medications. MedIndex-1 calculates the sum of the ratios of prescribed doses to standard doses, weighted by drug class. MedIndex-2 multiplies this sum by the number of medications, thereby capturing both the dosage and the number of prescribed medications. We calculated the mean of MedIndex-2 at each time point. The figure shows that MedIndex-2 decreased over time, indicating a reduced need for antihypertensive medications.

Supplementary Figure S4 eGFR. Change in eGFR post electro-hydraulic acoustic therapy treatment. Serum creatinine was taken at every timepoint from baseline to 48 weeks post treatment. Serum creatinine was used to calculate eGFR using CKD- EPI 2021 equation. In figure 4 we see a delta (change) between all time points and baseline.

## Data Availability

The raw data supporting the conclusions of this article will be made available by the authors, without undue reservation.

## Supplementary material

Supplementary Figure S1 Study design. Participants underwent a screening process to assess eligibility, followed by baseline evaluation prior to intervention. Eligible participants received six sessions of electro-hydraulic acoustic therapies. The end of treatment evaluation was conducted shortly after the last therapy session. Subsequently, follow-up assessments were scheduled at 4-, 12-, 24-, and 48-weeks post-treatment to monitor outcomes over time. In all follow-ups, office and unattended office blood pressure was measured. In every follow-up laboratory blood and urine samples were taken to evaluate eGFR and uPCR. At 12- and 48-weeks post treatment ambulatory blood pressure monitoring was conducted.

Supplementary Figure S2 Study recruitment flowchart. Flowchart depicting the recruitment and follow-up process of recruiting study participants. Of 24 patients screened, 21 consented to participate, while 3 failed screening. One patient revoked consent, leaving 20 who completed all eHAT assessments. Of these, 4 dropped out during follow-ups, resulting in 16 completing all follow-ups. One patient was removed due to errors in blood pressure (BP) readings, yielding 15 patients with complete and usable results.

Supplementary Figure S3 Medindex. Change in MedIndex-2 post electro-hydraulic acoustic therapy treatment: baseline to 48-week follow-up. MedIndex is a method used to quantify the use of antihypertensive medications. MedIndex-1 calculates the sum of the ratios of prescribed doses to standard doses, weighted by drug class. MedIndex-2 multiplies this sum by the number of medications, thereby capturing both the dosage and the number of prescribed medications. We calculated the mean of MedIndex-2 at each time point. The figure shows that MedIndex-2 decreased over time, indicating a reduced need for antihypertensive medications.

Supplementary Figure S4 eGFR. Change in eGFR post electro-hydraulic acoustic therapy treatment. Serum creatinine was taken at every timepoint from baseline to 48 weeks post treatment. Serum creatinine was used to calculate eGFR using CKD- EPI 2021 equation. In figure 4 we see a delta (change) between all time points and baseline.

## References

[1] Gupta A Nagaraju SP Bhojaraja MV Swaminathan SM Mohan PB. Hypertension in chronic kidney disease: an update on diagnosis and management. South Med J. (2023) 116(2):237–44. 10.14423/SMJ.0000000000001516 36724542

[2] Whelton PK Carey RM Aronow WS Casey DE Collins KJ Dennison Himmelfarb C 2017 ACC/AHA/AAPA/ABC/ACPM/AGS/APhA/ASH/ASPC/NMA/PCNA guideline for the prevention, detection, evaluation, and management of high blood pressure in adults: a report of the American College of Cardiology/American Heart Association task force on clinical practice guidelines. Circulation. (2018) 138(17):e484–594. 10.1161/CIR.0000000000000596 30354654

[3] Adjeroh L Brothers T Shawwa K Ikram M Al-Mamun MA. The association between polypharmacy and health-related quality of life among non-dialysis chronic kidney disease patients. PLoS One. (2023) 18(11):e0293912. 10.1371/journal.pone.0293912 37956162 PMC10642842

[4] Schmitt KE Edie CF Laflam P Simbartl LA Thakar CV. Adherence to antihypertensive agents and blood pressure control in chronic kidney disease. Am J Nephrol. (2010) 32(6):541–8. 10.1159/000321688 21042012

[5] Mancia G Kreutz R Brunström M Burnier M Grassi G Januszewicz A 2023 ESH guidelines for the management of arterial hypertension the task force for the management of arterial hypertension of the European Society of hypertension: endorsed by the international society of hypertension (ISH) and the European Renal Association (ERA). J Hypertens. (2023) 41(12):1874–2071. 10.1097/HJH.0000000000003480 37345492

[6] Wallbach M Born E Kämpfer D Lüders S Müller GA Wachter R Long-term effects of baroreflex activation therapy: 2-year follow-up data of the BAT neo system. Clin Res Cardiol. (2020) 109(4):513–22. 10.1007/s00392-019-01536-5 31388741

[7] Kalarus Z Merkely B Neužil P Grabowski M Mitkowski P Marinskis G Pacemaker-based cardiac neuromodulation therapy in patients with hypertension: a pilot study. J Am Heart Assoc. (2021) 10(16):e020492. 10.1161/JAHA.120.020492 34387126 PMC8475046

[8] Tolu-Akinnawo O Ray DN Awosanya T Nzerue C Okafor H. Hypertension and device-based therapies for resistant hypertension: an up-to-date review. Cureus. (2024) 16(8):e66304. 10.7759/cureus.66304 39108770 PMC11302934

[9] Zhang X Yan X Wang C Tang T Chai Y. The dose-effect relationship in extracorporeal shock wave therapy: the optimal parameter for extracorporeal shock wave therapy. J Surg Res. (2014) 186(1):484–92. 10.1016/j.jss.2013.08.013 24035231

[10] Ciampa AR de Prati AC Amelio E Cavalieri E Persichini T Colasanti M Nitric oxide mediates anti-inflammatory action of extracorporeal shock waves. FEBS Lett. (2005) 579(30):6839–45. 10.1016/j.febslet.2005.11.023 16325181

[11] Mariotto S Cavalieri E Amelio E Ciampa AR de Prati AC Marlinghaus E Extracorporeal shock waves: from lithotripsy to anti-inflammatory action by NO production. Nitric Oxide Biol Chem. (2005) 12(2):89–96. 10.1016/j.niox.2004.12.005 15740982

[12] Ogoyama Y Kario K. Differences in the effectiveness and safety of different renal denervation devices. Hypertens Res. (2024) 47(10):2678–84. 10.1038/s41440-024-01801-9 39014117

[13] Inker LA Eneanya ND Coresh J Tighiouart H Wang D Sang Y New creatinine- and cystatin C-based equations to estimate GFR without race. N Engl J Med. (2021) 385(19):1737–49. 10.1056/NEJMoa2102953 34554658 PMC8822996

[14] Wan SH Hart M Hajjar I. A novel measurement index for antihypertensive medication burden and its use. Hypertens. (2009) 54(5):e135–136. 10.1161/HYPERTENSIONAHA.109.140681 19805634

[15] Thomas G Xie D Chen HY Anderson AH Appel LJ Bodana S Prevalence and prognostic significance of apparent treatment resistant hypertension in chronic kidney disease: report from the chronic renal insufficiency cohort study. Hypertens. (2016) 67(2):387–96. 10.1161/HYPERTENSIONAHA.115.06487 PMC4713320 26711738

[16] Kidney Disease: Improving Global Outcomes (KDIGO) CKD Work Group. KDIGO 2024 clinical practice guideline for the evaluation and management of chronic kidney disease. Kidney Int. (2024) 105(4S):S117–314. 10.1016/j.kint.2023.10.018 38490803

[17] Poulter NR Borghi C Parati G Pathak A Toli D Williams B Medication adherence in hypertension. J Hypertens. (2020) 38(4):579–87. 10.1097/HJH.0000000000002294 31834123

[18] Chan RJ Helmeczi W Hiremath SS. Revisiting resistant hypertension: a comprehensive review. Intern Med J. (2023) 53(10):1739–51. 10.1111/imj.16189 37493367

[19] Azizi M Sanghvi K Saxena M Gosse P Reilly JP Levy T Ultrasound renal denervation for hypertension resistant to a triple medication pill (RADIANCE-HTN TRIO): a randomised, multicentre, single-blind, sham-controlled trial. Lancet Lond Engl. (2021) 397(10293):2476–86. 10.1016/S0140-6736(21)00788-1 34010611

[20] Kandzari DE Böhm M Mahfoud F Townsend RR Weber MA Pocock S Effect of renal denervation on blood pressure in the presence of antihypertensive drugs: 6-month efficacy and safety results from the SPYRAL HTN-ON MED proof-of-concept randomised trial. Lancet Lond Engl. (2018) 391(10137):2346–55. 10.1016/S0140-6736(18)30951-6 29803589

[21] Zhang X Krier JD Amador Carrascal C Greenleaf JF Ebrahimi B Hedayat AF Low-energy shockwave therapy improves ischemic kidney microcirculation. J Am Soc Nephrol JASN. (2016) 27(12):3715–24. 10.1681/ASN.2015060704 27297945 PMC5118474

[22] Bhatt DL Vaduganathan M Kandzari DE Leon MB Rocha-Singh K Townsend RR Long-term outcomes after catheter-based renal artery denervation for resistant hypertension: final follow-up of the randomised SYMPLICITY HTN-3 trial. Lancet Lond Engl. (2022) 400(10361):1405–16. 10.1016/S0140-6736(22)01787-1 36130612

[23] Mahfoud F Kandzari DE Kario K Townsend RR Weber MA Schmieder RE Long-term efficacy and safety of renal denervation in the presence of antihypertensive drugs (SPYRAL HTN-ON MED): a randomised, sham-controlled trial. Lancet Lond Engl. (2022) 399(10333):1401–10. 10.1016/S0140-6736(22)00455-X 35390320

[24] Hausdorf J Lemmens MM Heck KDW Grolms N Korr H Kertschanska S Selective loss of unmyelinated nerve fibers after extracorporeal shockwave application to the musculoskeletal system. Neuroscience. (2008) 155(1):138–44. 10.1016/j.neuroscience.2008.03.062 18579315

[25] Soranna D Zambon A Corrao G Zanchetti A Parati G Mancia G. Different effects of antihypertensive treatment on office and ambulatory blood pressure: a meta-analysis. J Hypertens. (2019) 37(3):467–75. 10.1097/HJH.0000000000001914 30234773

[26] Hemmelgarn BR Manns BJ Lloyd A James MT Klarenbach S Quinn RR Relation between kidney function, proteinuria, and adverse outcomes. JAMA. (2010) 303(5):423–9. 10.1001/jama.2010.39 20124537

[27] Vestersager SV Skov-Jeppesen SM Yderstraede KB Bistrup C Jensen BL Lund L. Low-intensity extracorporeal shockwave therapy in patients with diabetic kidney disease: a matched cohort study. Int Urol Nephrol. (2025) 57(7):2245–53. 10.1007/s11255-025-04379-4 39934556 PMC12167320

