# Supplementary figures and images for: Feasibility and safety of electrohydraulic acoustic therapy for treatment of hypertension in patients with chronic kidney disease
Source: Front Med Technol. 2026 Mar 3;8:1735319. doi: 10.3389/fmedt.2026.1735319 (PMC12992220; doi:10.3389/fmedt.2026.1735319)

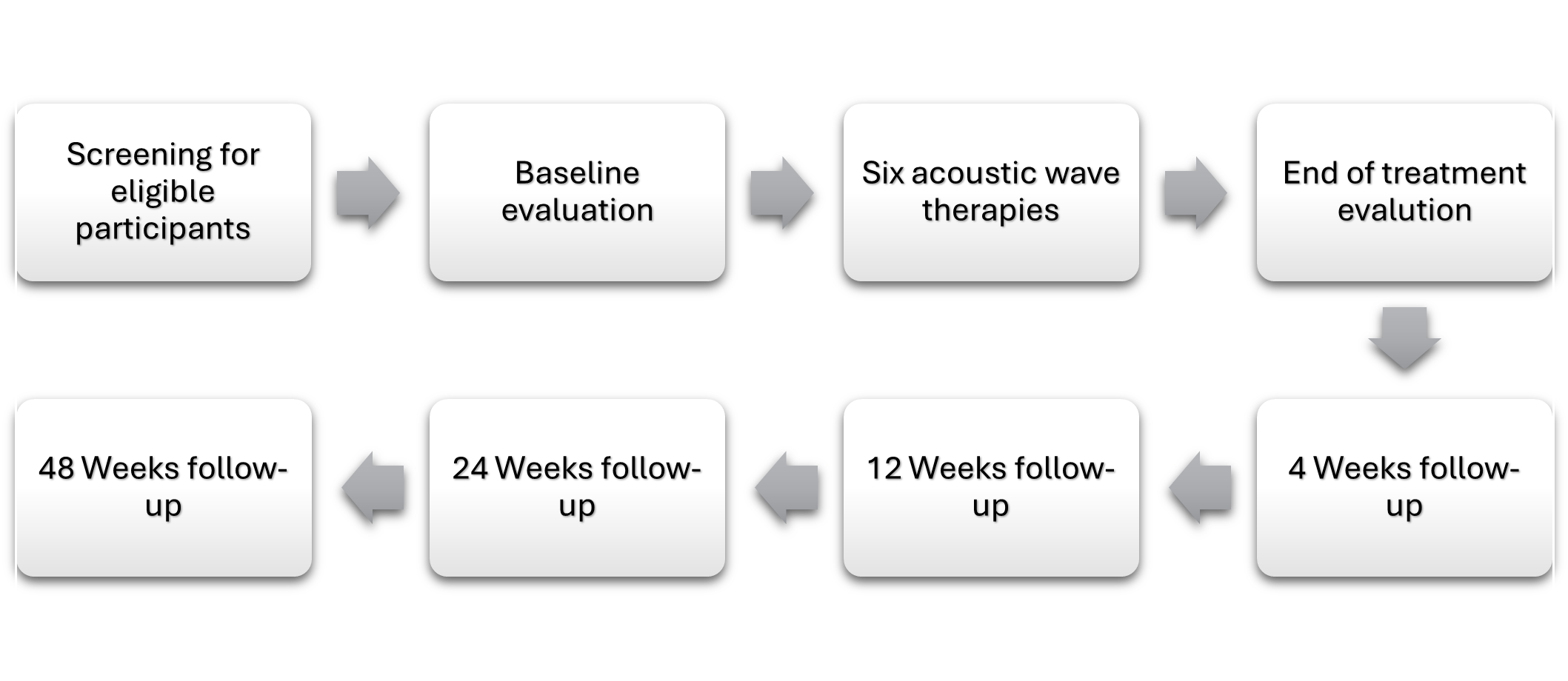

Supplement: Supplementary Figure S1 — Study design. Participants underwent a screening process to assess eligibility, followed by baseline evaluation prior to intervention. Eligible participants received six sessions of electro-hydraulic acoustic therapies. The end of treatment evaluation was conducted shortly after the last therapy session. Subsequently, follow-up assessments were scheduled at 4-, 12-, 24-, and 48-weeks post-treatment to monitor outcomes over time. In all follow-ups, office and unattended office blood pressure was measured. In every follow-up laboratory blood and urine samples were taken to evaluate eGFR and uPCR. At 12- and 48-weeks post treatment ambulatory blood pressure monitoring was conducted. [file Image1.tif]

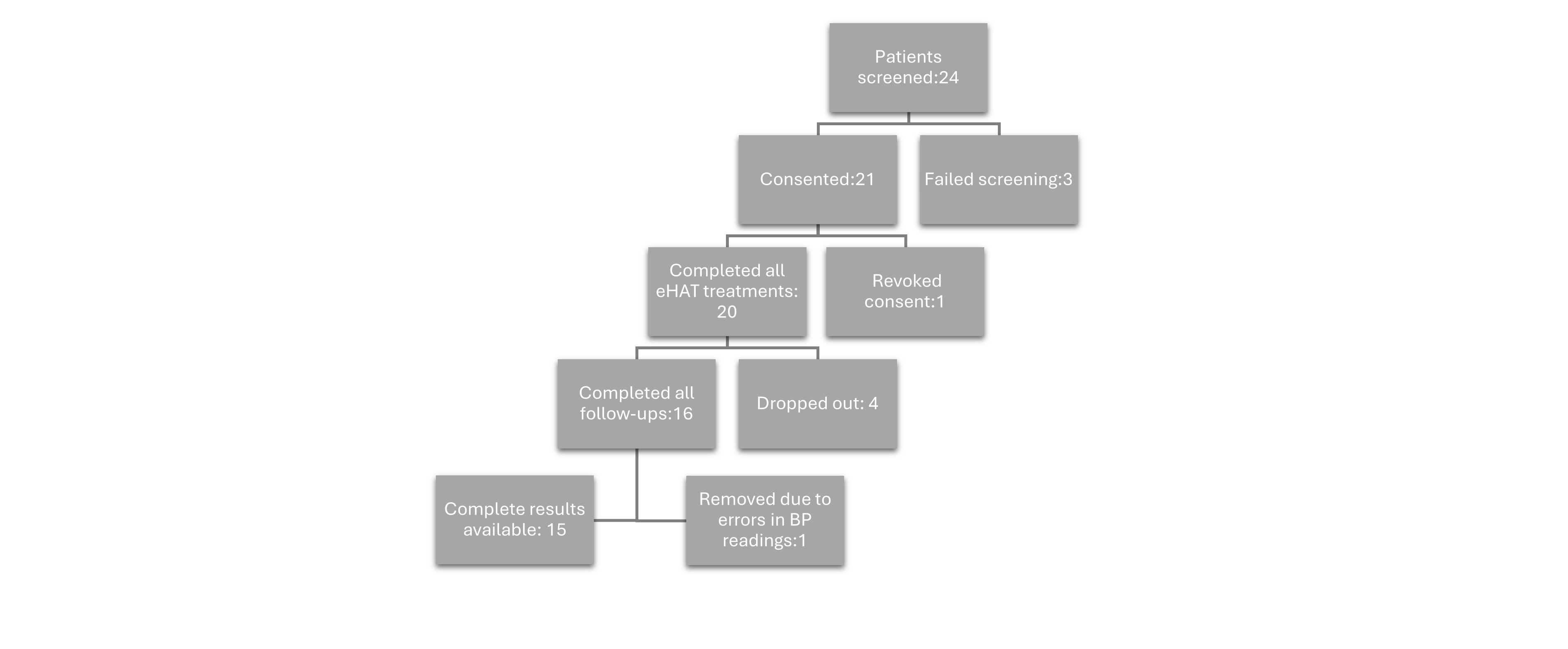

Supplement: Supplementary Figure S2 — Study recruitment flowchart. Flowchart depicting the recruitment and follow-up process of recruiting study participants. Of 24 patients screened, 21 consented to participate, while 3 failed screening. One patient revoked consent, leaving 20 who completed all eHAT assessments. Of these, 4 dropped out during follow-ups, resulting in 16 completing all follow-ups. One patient was removed due to errors in blood pressure (BP) readings, yielding 15 patients with complete and usable results. [file Image2.tif]

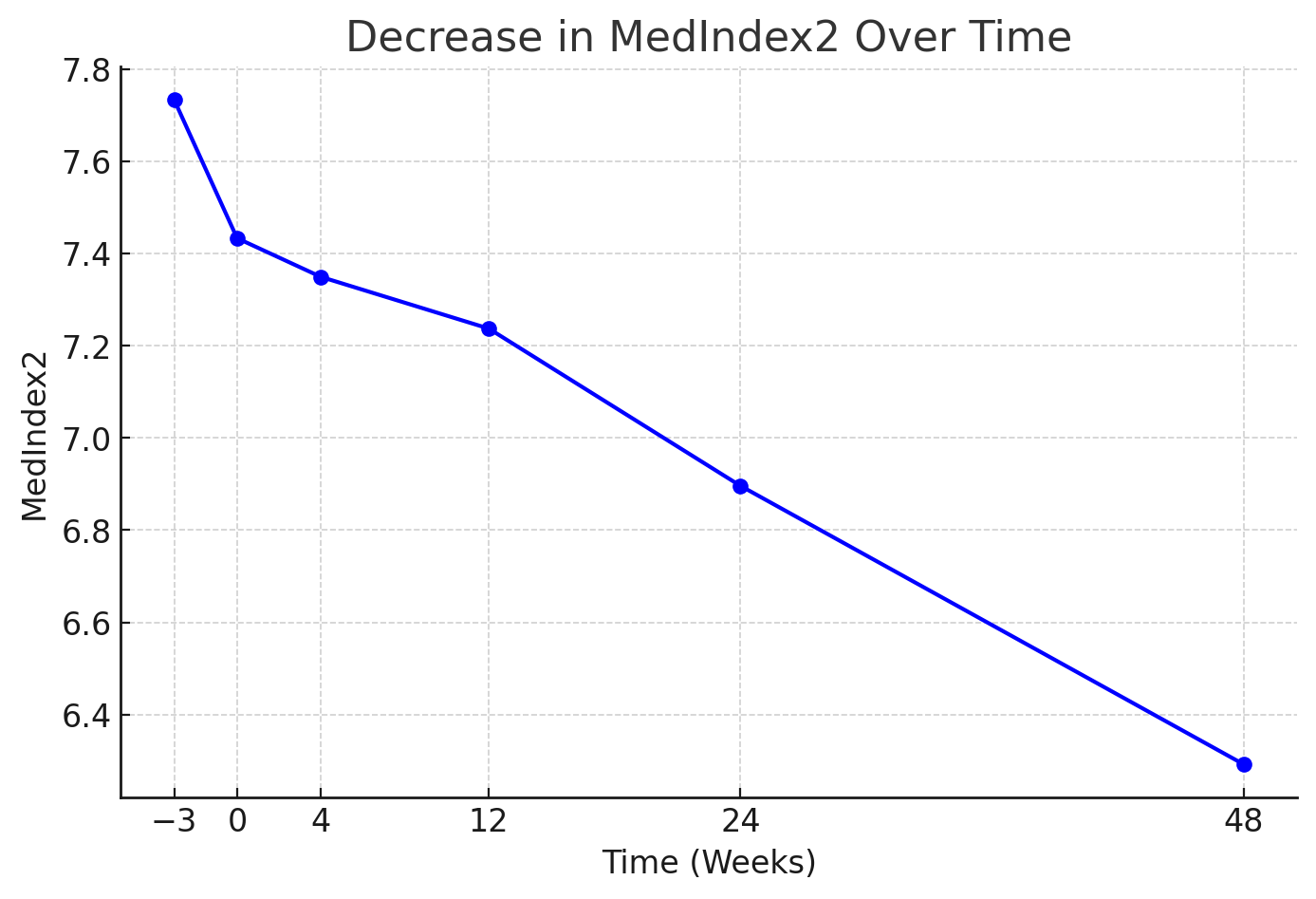

Supplement: Supplementary Figure S3 — Medindex. Change in MedIndex-2 post electro-hydraulic acoustic therapy treatment: baseline to 48-week follow-up. MedIndex is a method used to quantify the use of antihypertensive medications. MedIndex-1 calculates the sum of the ratios of prescribed doses to standard doses, weighted by drug class. MedIndex-2 multiplies this sum by the number of medications, thereby capturing both the dosage and the number of prescribed medications. We calculated the mean of MedIndex-2 at each time point. The figure shows that MedIndex-2 decreased over time, indicating a reduced need for antihypertensive medications. [file Image3.tif]

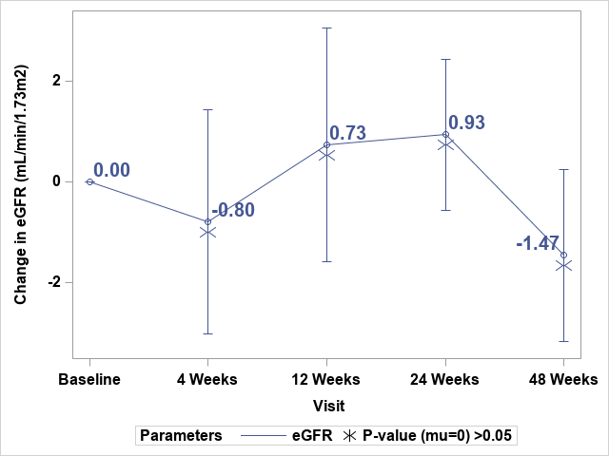

Supplement: Supplementary Figure S4 — eGFR. Change in eGFR post electro-hydraulic acoustic therapy treatment. Serum creatinine was taken at every timepoint from baseline to 48 weeks post treatment. Serum creatinine was used to calculate eGFR using CKD- EPI 2021 equation. In figure 4 we see a delta (change) between all time points and baseline. [file Image4.tif]
