# Supplementary material for: Feasibility and safety of electrohydraulic acoustic therapy for treatment of hypertension in patients with chronic kidney disease
Source: Front Med Technol. 2026 Mar 3;8:1735319. doi: 10.3389/fmedt.2026.1735319 (PMC12992220; doi:10.3389/fmedt.2026.1735319)
Supplement: Supplementary file 5 [file Table1.docx]

**Supplemental table 1.** Change in MedIndex post electro-hydraulic acoustic therapy treatment: baseline to 48-week follow-up.

| **Participant** | **Baseline** | **EOT** | **4 weeks** | **12 weeks** | **24 weeks** | **48 weeks** |
| --- | --- | --- | --- | --- | --- | --- |
| **Pt. 1** | 0,00 | 0,00 | 0,00 | 0,00 | 0,00 | 0,00 |
| **Pt. 3** | 20,63 | 20,63 | 23,63 | 24,38 | 24,38 | 15,31 |
| **Pt. 4** | 1,88 | 1,88 | 1,88 | 1,88 | 1,88 | 1,88 |
| **Pt. 5** | 2,50 | 2,50 | 2,50 | 2,50 | 2,50 | 2,50 |
| **Pt. 6** | 3,50 | 3,50 | 1,75 | 1,75 | 1,75 | 1,75 |
| **Pt. 8** | 4,43 | 4,43 | 4,43 | 4,43 | 1,88 | 1,88 |
| **Pt. 9** | 9,75 | 9,75 | 9,75 | 9,75 | 9,75 | 9,75 |
| **Pt. 10** | 11,25 | 11,25 | 11,25 | 11,25 | 15,94 | 15,94 |
| **Pt. 12** | 5,00 | 5,00 | 2,50 | 5,00 | 5,00 | 5,00 |
| **Pt. 14** | 12,19 | 12,19 | 12,19 | 7,25 | 7,75 | 7,75 |
| **Pt. 15** | 14,38 | 14,38 | 14,38 | 14,38 | 10,00 | 10,00 |
| **Pt. 16** | 3,75 | 2,25 | 2,25 | 2,25 | 2,25 | 2,25 |
| **Pt. 18** | 12,00 | 12,00 | 12,00 | 12,00 | 11,25 | 11,25 |
| **Pt. 21** | 3,50 | 3,50 | 3,50 | 3,50 | 3,50 | 3,50 |
| **Pt. 24** | 11,25 | 8,25 | 8,25 | 8,25 | 5,63 | 5,63 |
| **Average Medindex** | **7,733** | **7,433** | **7,349** | **7,237** | **6,896** | **6,292** |

|  |
| --- |

EOT= end of treatment
